# Exciton-related nonlinear optical properties in cylindrical quantum dots with asymmetric axial potential: combined effects of hydrostatic pressure, intense laser field, and applied electric field

**DOI:** 10.1186/1556-276X-7-508

**Published:** 2012-09-12

**Authors:** Alejandro Zapata, Ruben E Acosta, Miguel E Mora-Ramos, Carlos A Duque

**Affiliations:** 1 Instituto de Física, Universidad de Antioquia, A.A. 1226, Medellín, Colombia; 2 Facultad de Ciencias, Universidad Autónoma del Estado de Morelos, Av. Universidad 1001, CP 62209, Cuernavaca, Morelos, Mexico

**Keywords:** Exciton binding energy, GaAs-Ga_1−*x*_Al_*x*_As cylindrical quantum dot, Effective mass approximation, 78.67.De; 71.55.Eq; 32.10.Dk

## Abstract

The exciton binding energy of an asymmetrical GaAs-Ga_1−*x*_Al_*x*_As cylindrical quantum dot is studied with the use of the effective mass approximation and a variational calculation procedure. The influence on this quantity of the application of a direct-current electric field along the growth direction of the cylinder, together with that of an intense laser field, is particularly considered. The resulting states are used to calculate the exciton-related nonlinear optical absorption and optical rectification, whose corresponding resonant peaks are reported as functions of the external probes, the quantum dot dimensions, and the aluminum molar fraction in the potential barrier regions.

## Background

Exciton states in quantum dot (QD) systems are relevant to the understanding of a number of their optical and electronic features. There have been a significant number of works dealing with excitonic effects in these nanodimensional structures (see, for instance, [1]). Among the external probes affecting the spectrum of localized states in semiconducting nanostructures, we can mention the electric field. There is a work from Peter and Lakshminarayana on the influence of the electric field on donor binding energies in QDs with parabolical, spherical, and rectangular confinement [2]. Besides, the asymmetric potential quantum well (QW) configuration can lead to the enhancement of the interband oscillator strength through the obtention of larger values of the dipole moments of the optical transitions. It is possible to predict the obtention of important exciton-related nonlinear optical responses in these confined semiconducting structures. We can mention some theoretical studies regarding the optical nonlinearities [3,4].

The application of intense laser fields (ILFs) to low-dimensional semiconductor systems has allowed the appearance of new and interesting features in their electronic structures. One of them is the ILF-induced transition to a double-QW configuration in an otherwise single-well heterostructure [5]. This phenomenon occurs when the value of the so-called laser-dressing parameter [6] becomes larger than the half-width of the QW. The mathematical description goes through deriving a modified form of the confining potential energy function [7]. Niculescu and Burileanu have put forward calculations on shallow impurity states in QW wires and QWs of different geometries, combining the ILF effects and the application of static magnetic and electric fields [8]. We have reported on the influence of the laser-induced transition from single- to double-well potential on impurity states in a GaAs-based QW [9], whereas the optical response of semiconducting nanostructures subject to the radiation of high-intensity laser fields has also been a matter of some studies in the last few years (see, for instance, [10-12]).

With this motivation, in this work, we investigate the properties of the exciton-related nonlinear optical absorption (NOA) and nonlinear optical rectification (NOR) in cylindrical GaAs-Ga_1−*x*_Al_*x*_As QDs under ILFs, direct-current (dc) electric fields, and hydrostatic pressure.

## Methods

### Theoretical framework

In what follows, we shall express energies in effective Rydbergs $\left(R_{0}=\frac{\mu \;e^{4}}{2\;\hbar^{2}\;\varepsilon^{2}}\right)$ and lengths in effective Bohr radii $\left(a_{0}=\frac{\hbar^{2}\;\varepsilon }{\mu \;e^{2}}\right)$. According to the model proposed by Le Goff and Stébé, within the effective mass approximation, the Hamiltonian for exciton states in a cylindrical GaAs-Ga_1−*x*_Al_*x*_As QD under in-growth-direction applied electric field (*F*) is given by [1]

(1) $$\;\begin{array}{l} H=\underset{i=\text{e,h}}{\sum }\left\{\;-\frac{\mu }{m_{i}^{*}}\left[\;\frac{\partial^{2}}{\partial \rho_{i}^{2}}\;+\;\frac{1}{\rho_{i}}\frac{\partial }{\partial \rho_{i}}+\frac{\rho^{2}\pm (\rho_{\text{e}}^{2}-\rho_{\text{h}}^{2})}{\rho \rho_{i}}\frac{\partial^{2}}{\partial \rho \partial \rho_{i}}\right]\right)\\ \;-\left(\frac{\mu }{m_{i}^{*}}\frac{\partial^{2}}{\partial z_{i}^{2}}\;+\;V_{i}\left(\rho_{i},z_{i}\right)\pm e\;F\;z_{i}\;\right\}-\left[\;\frac{\partial^{2}}{\partial \rho^{2}}\;+\;\frac{1}{\rho }\frac{\partial }{\partial \rho }\;\right]\;-\;\frac{2}{r}, \end{array}$$

where $r=\sqrt{\rho^{2}+{\left(z_{\text{e}}-z_{\text{h}}\right)}^{2}}$, $\rho =|\vec{\rho_{\text{e}}}-\vec{\rho_{\text{h}}}|$, and ± stands for electrons and holes; $m_{\text{e}}^{*}$ ( $m_{\text{h}}^{*}$) labels electron (hole) effective mass, while *μ* is the electron-hole reduced mass, *e* is the electron charge, *ε* is the GaAs static dielectric constant, and *V*_e_(*ρ*_e_*z*_e_)*V*_h_(*ρ*_h_*z*_h_)] is the QD confinement potential function for the electron (hole) carrier. We assume that the applied electric field is oriented along (0, 0, −*z*).

Our model considers a QD of cylindrical shape with radius *R* and height *L*. For the confinement potential of the carriers, we have considered infinite and finite confinement potentials in the *ρ*- and *z*-directions, respectively. In such an approximation, it is valid to consider the separation $V_{i}(\rho_{i},z_{i})=V_{i}^{1}(\rho_{i})+V_{i}^{2}(z_{i})$ (*i *= e,h). Here, $V_{i}^{1}(\rho_{i})=0$ for *ρ*_*i *_≤* R* and $V_{i}^{1}(\rho_{i})\to \infty$ for *ρ*_*i *_>* R*. In the case of the *z*-dependent asymmetric confinement, we have $V_{i}^{2}(z_{i})=0$ for |*z*_*i*_| ≤* L*/2, $V_{i}^{2}(z_{i})=V_{0}^{i}(x_{\text{Al}}^{1})$ when *z*_*i *_≤ −*L*/2, and $V_{i}^{2}(z_{i})=V_{0}^{i}(x_{\text{Al}}^{2})$ when *z*_*i*_≥ + *L*/2. We use the value of the aluminum contents, ( $x_{\text{Al}}^{1}$, $x_{\text{Al}}^{2}$), in the confining barriers to monitor their corresponding heights. For electrons (holes), the axial confinement potential barrier heights $V_{0}^{\text{e}}$ ( $V_{0}^{\text{h}}$) are obtained from the 60% (40%) of the bandgap between the well (GaAs) and barrier (Ga_1−*x*_Al_*x*_As) materials ( $\Delta E_{g}^{0}=(1,155\;x+370\;x^{2}$) meV).

In order to obtain the exciton eigenfunctions, and the corresponding energies (*E*), we adopt a variational scheme [13] which consists minimizing the functional *E*(*Ψ*)=〈*Ψ*|*H*|*Ψ*〉 by using the trial wave function, *Ψ*, as [1]

(2) $$\begin{array}{lll} \Psi_{n_{e},n_{h}}({\vec{r}}_{\text{e}},{\vec{r}}_{\text{h}}) & =N_{n_{e},n_{h}}\;\Upsilon_{n_{e},n_{h}}\; & \;\\ & \;\times (\rho_{\text{e}},\rho_{\text{h}},z_{\text{e}},z_{\text{h}})e^{-\alpha_{n_{e},n_{h}}\rho -\beta_{n_{e},n_{h}}{\left(z_{e}-z_{h}\right)}^{2}},\; \end{array}$$

where $N_{n_{e},n_{h}}$ is the normalization constant, $\alpha_{n_{e},n_{h}}$ and $\beta_{n_{e},n_{h}}$ are variational parameters, and $\Upsilon_{n_{e},n_{h}}\;(\rho_{\text{e}},\rho_{\text{h}},z_{\text{e}},z_{\text{h}})=F(\rho_{\text{e}})\;F(\rho_{\text{h}})\;g_{n_{e}}(z_{\text{e}})\;g_{n_{h}}(z_{\text{h}})$ is the eigenfunction of the Hamiltonian in Equation 1 without the Coulomb term at the right-hand side. The integer numbers *n*_*e*_ and *n*_*h *_identify the quantized states for both the electron and the hole, respectively, associated with the confinement along the *z*-direction. The uncorrelated radial wave functions for single particles are given by *F*(*ρ*_*i*_) =* J*_0_(*θ*_0_*ρ*_*i*_/*R*), where *J*_0_ represents the Bessel function of the zeroth order and *θ*_0_ = 2.4048 is its first root on the real axis. The way of obtaining the uncorrelated axial wave functions for single particles relies in the method developed by Xia and Fan [14]. The energy of the exciton, $E^{n_{e},n_{h}}$, is calculated by minimizing the Hamiltonian with the trial wave function. The exciton binding energy ( $E_{\text{b}}^{n_{e},n_{h}}$) is obtained from the definition $E_{\text{b}}^{n_{e},n_{h}}=E_{0}^{n_{e},n_{h}}-E^{n_{e},n_{h}}$, where $E_{0}^{n_{e},n_{h}}$ is the eigenvalue associated to $\Upsilon_{n_{e},n_{h}}(\rho_{\text{e}},\rho_{\text{h}},z_{\text{e}},z_{\text{h}})$. In the present work, we are interested in the two lowest excitonic states: *ϕ*_1_≡*Ψ*_1,1_ and *ϕ*_2_≡*Ψ*_2,1_, with energies $E^{1,1}\equiv E_{1}$ and $E^{2,1}\equiv E_{2}$, respectively.

Looking to include the nonresonant intense laser effects (with the choice of the polarization of laser radiation to be parallel to the *z*-direction), we have followed the Floquet method. To briefly summarize the outcome of such a procedure, it is possible to say that the information regarding laser influence will appear in the *z*-dependent part of the confinement potential in Equation 1 by substituting $V_{i}^{2}(z_{i})\to \langle V_{i}^{2}\rangle (z_{i},\alpha_{0i})$[5,6,15,16]. In that expression, the quantity $\alpha_{0i}=(eA_{0})/(m_{i}^{*},c\varpi )=(I^{1/2}/\varpi^{2})(e/m_{i}^{*}){\left(8\Pi /c\right)}^{1/2}$ is the laser-dressing parameter. Here, I and *ϖ *are, respectively, the average intensity and the frequency of the laser field wave. *A*_0_ represents the amplitude of the vector potential of the incident radiation. In the case of the Coulomb interaction, the last term in Equation 1 must be replaced as

(3) $$\frac{2}{r}\to \frac{1}{\sqrt{\rho^{2}+{\left(z_{\text{eh}}+\alpha_{0}\right)}^{2}}}+\frac{1}{\sqrt{\rho^{2}+{\left(z_{\text{eh}}-\alpha_{0}\right)}^{2}}}$$

with *z*_eh_ =* z*_e_−*z*_h_ and $\alpha_{0}=(e\;A_{0})/(\mu^{*}\;c\;\varpi )$.

After the energies and the corresponding envelope wave functions are obtained, the magnitude of the resonant peaks of the $E_{1}⇆E_{2}$ exciton-related NOA and NOR coefficients can be derived under a density matrix approach. They are given, respectively, by [17]

(4) $$\;\;\;\;\alpha_{\text{max}}=\frac{e^{2}\;N\;\omega_{21}\;M_{21}^{2}\;T_{2}}{\hbar \;\varepsilon_{0}\;c\;n}$$

and

(5) $$\chi_{0,\text{max}}=\frac{2\;|e{|}^{3}\;N\;M_{21}^{2}\;|M_{22}-M_{11}|\;T_{1}\;T_{2}}{\varepsilon_{0}\;\hbar^{2}}\;,$$

where $M_{\mathrm{ij}}=<\phi_{i}|z_{\text{e}}-z_{\text{h}}|\phi_{j}>$. Also, *ε*_0_ is the vacuum permittivity, *n* is the refractive index of the QD material, *N* is the electron density in QD, and $\omega_{21}=(E_{2}-E_{1})/\hbar$. The quantities *T*_1,2_represent the lifetimes that associate with the damping in the system. Moreover, the hydrostatic pressure effects are introduced via the pressure dependence of the effective masses and the dielectric constant. In the calculations, we have used $m_{\text{e}}^{*}={\left[1+15,020/E_{g}+7,510/(E_{g}+341)\right]}^{-1}$, $m_{\text{h}}^{*}=0.34-0.1\times 10^{-3}\;P$, and $\varepsilon =12.7\;\exp(-1.67\times 10^{-3}\;P)$. Here, *E*_*g *_= (1,519 + 10.7 *P*) meV, *P* is the hydrostatic pressure (in kbar), and *m*_0_is the free electron mass [18].

## Results and discussion

Let us now go back to giving the energy values in units of millielectron volts and the lengths in nanometers. The calculations use *T*_1_ = 1 ps, *T*_2_ = 0.2 ps, and *N *= 3×10^22^m^−3^[17,19]. We have chosen to report the calculated quantities as functions of the Al molar fraction in the left-hand barrier. The composition of aluminum in the right-hand potential barrier remains fixed at $x_{\text{Al}}^{2}=0.33$

**Figure 1:**
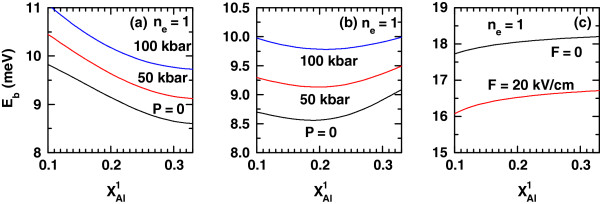
**Binding energy of 1*s*-like heavy-hole exciton state as a function of left-hand- potential-barrier aluminum concentration (**$x_{\text{Al}}^{1}$**).** The results are for the ground exciton state (*n*_*e *_= 1 and *n*_*h *_= 1) with $x_{\text{Al}}^{2}=0.33$, *L *= 15 nm, and *R *= 10 nm. (**a**, **b**) Several values of the hydrostatic pressure with different setups of the applied electric field for *α*_0_(*P*) = 3*L*(*P*)/4: (a) *F *= 0 kV/cm and (b) *F *= 20 kV/cm, have been considered. (**c**) The results are for two values of the applied electric field with *P *= 0 and *α*_0_ = 0.

**Figure 2:**
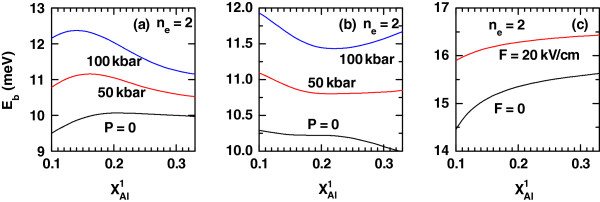
**The same results presented in Figure**1**but for the case of first-excited exciton state.** The system has identical geometric-plus-external-probe configurations ( *n*_*e *_= 2 and *n*_*h *_= 1).

Figure 1 shows the dependence of the ground (*n*_*e *_= 1 and *n*_*h *_= 1) exciton binding energy as a result of the variation of the left-hand-barrier Al composition $x_{\text{Al}}^{1}$. The results correspond to the geometric configuration: *L *= 15 nm; *R *= 10 nm. In Figure 1a,b, we have considered several values of the hydrostatic pressure with different setups of the dc electric field (*F *= 0 kV/cm (a); *F *= 20 kV/cm (b)), taking a constant value of the intense laser field parameter *α*_0_(0) = 3*L*(0)/4 = 11.3 nm. Figure 1c contains the results of $E_{\text{b}}(x_{\text{Al}}^{1})$ with *P *= 0 and *α*_0_ = 0, for two values of the applied dc electric field. Results for *E*_b_in a system with equal geometric and external configurations, but for the first-excited exciton state, are presented in Figure 2.

Let us first explain the results for *E*_b_of Figures 1c and 2c. In both figures, we notice an increasing variation of *E*_b_ as a result of the increment in $x_{\text{Al}}^{1}$. This happens with and without applied dc electric field and relates with the growing degree of the carrier confinement. Augmenting the Al molar fraction in the left-hand barrier leads to the effective deepening of the quantum potential well for electrons and holes. The electron (both the ground and the excited state) and hole wave functions become more spatially localized in the well region, and the expected electron-hole distance diminishes. This has the effect of strengthening the Coulombic interaction, with the observed growth in the binding energy.

The inversion in energy position of the curves with *F *= 0 and 20 kV/cm, seen when going from Figure 1c to Figure 2c is due to the dc-electric-field-induced linear decrement of the height of one of the potential barriers and the linear increase of the other. This, together with the inclination in the QW bottom position, makes the carriers to be less localized inside the well, displacing the ground-state density of probability towards one side of the system. This influence is opposite in sign for electrons and holes. The expected electron-hole distance augments and the Coulombic attraction weakens for nonzero *F*. In the case of the first-excited exciton complex, the effect of an augmenting dc field on the hole-confined level is the same, but the wave function of the conduction electron with *n*_*e *_= 2 is deformed in such a way that most of its probability density has a greater spatial coincidence with that of the hole in *n*_*h *_= 1. Thus, the quantity |〈*ϕ*_2_|*z*_e_−*z*_h_|*ϕ*_1_〉| will have a lower value, and the Coulombic attraction will be stronger.

**Figure 3:**
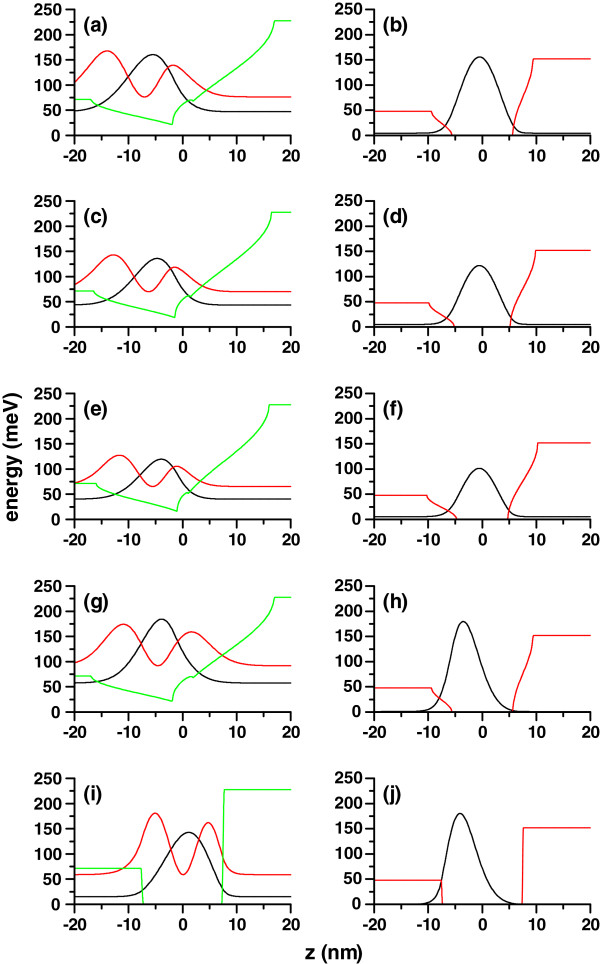
**Confining potential for electrons (left panel) and holes (right panel) for an electron-hole system (green lines).** The system is confined in a cylindrical quantum dot with a radius of 10 nm and a height of 15 nm with $x_{\text{Al}}^{1}=0.1$ and $x_{\text{Al}}^{2}=0.33$. The electron probability densities along the axial direction are shown for both the ground (black lines) and first-excited (red lines) states, and only the ground hole state is shown. Different configurations (*P*, *F*, *α*_0_) are considered: 0, 0, 11.3 nm (**a**, **b**); 50 kbar, 0, 11.3 nm (**c**, **d**); 100 kbar, 0, 11.3 nm (**e**, **f**); 0, 20 kV/cm, 11.3 nm (**g**, **h**); and 0, 20 kV/cm, 0 (**i**, **j**).

Figure 3 contains a picture of the confining potential for both electrons and holes for different geometric configurations - related with the intensity of the laser field. Schematic representations of the ground hole state and the ground and first-excited electron states are provided as well. The aim of this figure is to help understand the features of the binding energy depicted in Figures 1 and 2: 

1. By comparing Figures 1a and 2a, it becomes clear that for finite values of the hydrostatic pressure, the binding energy is larger in the excited state compared with the ground state ( $E_{\text{b}}^{2,1}>E_{\text{b}}^{1,1}$). This situation is contrary to the one observed in the case of *P *= 0 ( $E_{\text{b}}^{1,1}>E_{\text{b}}^{2,1}$). We can observe from Figure 3a,b, which corresponds to the case of *P*=0, that the density of probability of the electron excited state (*n*_*e *_= 2) is more extended along the structure. This bound (excited) state corresponds, in fact, to the infinite barrier system and not to our QW, but the electron ground state (*n*_*e *_= 1) is actually a confined state of the QW. Consequently, according to these arguments, $E_{\text{b}}^{1,1}>E_{\text{b}}^{2,1}$, given that the average electron-hole distance is smaller if the electron moves with the energy of the confined ground state. In Figure 3c,d, which is valid when *P *= 50 kbar (Figure 3e,f, valid for *P *= 100 kbar), it is possible to observe that the state with *n*_*e *_= 2 tends to become localized within the QW region; in fact, it is bound to the QW when *P *= 100 kbar, whereas the ground state remains with the same zero-pressure character. As a result, we obtain $E_{\text{b}}^{2,1}>E_{\text{b}}^{1,1}$.

2. The comparison between the results in Figures 1b and 2b yields clearly to the situation $E_{\text{b}}^{2,1}>E_{\text{b}}^{1,1}$. In Figure 3g,h, we are depicting the densities of probability that correspond to the electron and hole states involved in the formation of the two excitons. The application of an electric field will push the electron and hole wave functions to opposite directions in the case of both uncorrelated ground states with the consequent increment of the electron-hole distance and reduction of the binding energy. However, a very different situation is that involving the coupling of the ground hole state and the first-excited electron one, whose wave functions are displaced towards the same space region, decreasing the electron-hole distance and augmenting the corresponding exciton binding energy.

3. When we compare the results in Figures 1c and 2c, it can be clearly observed that the condition $E_{\text{b}}^{1,1}>E_{\text{b}}^{2,1}$ is always fulfilled. The zero-laser-field and zero-pressure case corresponds to the situation seen in Figure 3i,j. We can, therefore, extract the following information: Although the action of the electric field rises the proximity of the hole and first-excited electron wave functions and makes the wave functions of the hole and ground electron states to move further apart, it is a fact that under the particular conditions for the obtention of those results, one finds that $E_{\text{b}}^{1,1}>E_{\text{b}}^{2,1}$. This is a consequence of the strong confinement of the electron ground state within the QW region, spatially close to the maximum of the ground hole probability density, with really little influence of the electric field. Given that the first-excited electron state lies near the potential barrier edge, the application of an electric field largely modifies it, generating a bigger separation between the electron and the hole and a decrease of the binding energy.

Now, we come back to the discussion of Figures 1 and 2. Regarding the results of Figures 1a and 2a, we can say that the significant distinction in the potential profiles that confine electrons and heavy holes is a consequence of the important difference between the effective masses of both kinds of carriers. The potential profile for the conduction electrons, given their lightness, is strongly modified by the effect of the laser field, whereas the deformation of the heavy-hole valence band is smaller. The descent observed for $E_{\text{b}}(x_{\text{Al}}^{1})$ in Figure 1a is due, then, to the diminishing of the carrier confinement. Augmenting $x_{\text{Al}}^{1}$ produces a higher potential barrier at the left-hand side. This is reflected in a progressively more homogeneous raising of the QW bottom caused by the change in the laser field-induced change in the QW shape. Thus, the energy position of the electron ground state shifts upwards, and its density of probability spreads over a larger region. On the contrary, the hole state remains more localized when the left-hand barrier is higher, in spite of the laser-induced valence band bending deformation that narrows the QW’s lower part. This pushes the energy levels upwards, and larger values of the expected electron-hole distance are then obtained.

With hydrostatic pressure applied, the ground state-related exciton binding energy keeps decreasing along the whole range of the left-hand-barrier Al composition values. However, as we notice from Figure 1a, the starting and ending values of the curves are increasingly higher with *P*. There are two main reasons for this phenomenon: (1) The hydrostatic pressure induces growth in the electron effective mass, the magnitude of the conduction band ground-state energy becomes smaller, with an associated increment of the wave function confinement. This shifting effect on heavy holes is less pronounced, and the change of *m*_e_(*P*) becomes the dominant effect. As a result of this, there will be a reduction in the expected electron-hole distance, as well as an increase in the binding energy, for a given value of $x_{\text{Al}}^{1}$ if compared with the zero-pressure situation. (2) The dielectric constant is a decreasing function of the hydrostatic pressure. Hence, again fixing a value of the Al composition in the left-hand barrier, a larger value of the pressure will affect the Coulombic interaction in the system by increasing its magnitude. This latter effect is the most important.

Let us analyze now the same problem but for the first-excited exciton state when *F *= 0 (Figure 2a). The argument related with the pressure-induced changes in the effective masses and dielectric constant keeps its validity to justify the higher values of *E*_b_ for nonzero pressures compared with those of the *P *= 0 case. However, unlike the ground-state exciton, the first-excited binding energy is not a monotonically decreasing function of $x_{\text{Al}}^{1}$ over the range of values of this quantity considered. Considering first the zero-pressure regime, we observe a slight growth in *E*_b_ when the Al concentration in the left-hand barrier augments from 0 to approximately 0.18. Such an increment associates with the higher localization of the *n*_*e *_= 1 wave function inside the QW region due to the presence of higher confining barriers. If the value of $x_{\text{Al}}^{1}$ grows beyond that point, a very smoothly decreasing variation of *E*_b_(*P *= 0) will start, due to the loss in electron confinement, with the corresponding augmenting rate for the expected electron-hole distance. For finite pressures, we find the same growing behavior for the smaller values of the Al molar fraction, but the ulterior decrease is much more pronounced, resembling that exhibited by the exciton binding energy of the ground state. In this case, the explanation can be found in the pressure effect on the *n*_*e *_= 1 state localization.

The results presented in Figures 1b and 2b correspond to the variation of the exciton binding energy due to the increment in the Al composition in the left-hand confining barrier in the situation in which there is an additional applied dc electric field with the amplitude fixed to *F *= 20 kV/cm. The configuration includes the application of laser radiation with the intensity characterized by *α*_0_(*P*) = 3*L*(*P*)/4. The distribution of *E*_b_curve positions with the pressure value as a parameter is the same as in the zero-dc-field case and can be explained by the arguments above. The monotony of the curves is significantly different in this case. The ground-exciton binding energy as a function of $x_{\text{Al}}^{1}$ (Figure 1b) shows initially a decreasing behavior until the left-hand-barrier Al composition is approximately 0.18. Above this value, all curves change their variation to an increasing one. The reduction in *E*_b_ for the smaller values of the Al composition is mostly due to the increment in the expected electron-hole distance associated to the additional electron-hole polarization induced by the dc field. The subsequent growth in *E*_b_ relates with the effect of the higher left-hand barrier on the displaced electron wave function. A higher potential barrier repels the shifted probability density of the ground electron state away from the corresponding interface. This causes a reduction in the average electron-hole distance and the observed growth of *E*_b_ for the larger values of $x_{\text{Al}}^{1}$.

The variations of the first-excited exciton binding energy with respect to the increment in the left-hand-barrier Al alloy composition, considering three values of the hydrostatic pressure as parameters and the presence of intense laser and applied dc fields (with intensity *F*=20 kV/cm) also show a decreasing monotony when $x_{\text{Al}}^{1}$ augments in the region of its smaller values. The upper value for the decrease of *E*_b_ depends on the hydrostatic pressure. If *P *= 0, then the binding energy is an all the way decreasing function of the Al concentration. The increase in the expected electron-hole distance induced by the dc field - which is predominant for the lower alloy compositions - can be understood by remembering that the excited state is less affected by barrier repulsion. Augmenting the left-hand-barrier height - tending to convert the system into a symmetric QW - causes the pushing effect of the raising in the QW bottom to have a greater influence on the delocalization of the *n*_*e *_= 2 state, leading to the weakening of the Coulombic interaction. When the pressure goes up and reaches 50 kbar, there will be a slight growth in *E*_b_above the value of $x_{\text{Al}}^{1}$ at which the repulsive barrier interaction becomes predominant. The application of hydrostatic pressure augments the electron effective mass and the first-excited electron level occupies a lower energy position, thus granting a higher sensitivity to the barrier rising. If the pressure value is even bigger (*P *= 100 kbar), *E*_b_ recovers the increasing variation for larger values of the left-hand Al composition, as what happens for the ground exciton state.

**Figure 4:**
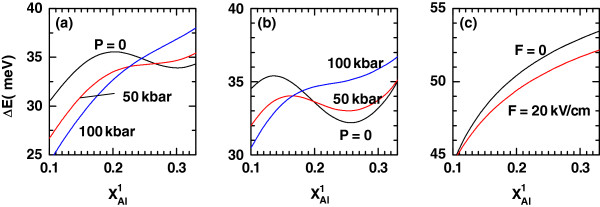
**The transition energy between the ground and first-excited exciton states in a cylindrical QD.** The asymmetrical axial potential configuration is a function of the aluminum concentration in the left-hand potential barrier ( $x_{\text{Al}}^{1}$). The results are for $x_{\text{Al}}^{2}=0.33$, *L *= 15 nm, and *R *= 10 nm. (**a**, **b**) Several values of the hydrostatic pressure with different setups of the applied electric field for values of the intense laser parameter *α*_0_(*P*) = 3*L*(*P*)/4 = 11.3 nm: (a) *F *= 0 kV/cm and (b) *F *= 20 kV/cm, have been considered. (**c**) The results appear for two values of the applied electric field with *P *= 0 and *α*_0_ = 0.

**Figure 5:**
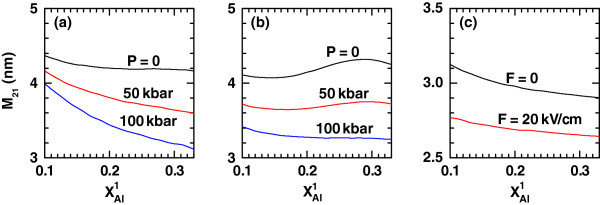
**The transition dipole matrix element between the ground and first-excited exciton states in a cylindrical QD.** The asymmetrical axial potential configuration is a function of the aluminum concentration in the left-hand potential barrier ( $x_{\text{Al}}^{1}$). The results are for $x_{\text{Al}}^{2}=0.33$, *L *= 15 nm, and *R *= 10 nm. (**a**, **b**) Several values of the hydrostatic pressure with different setups of the applied electric field for values of the intense laser parameter *α*_0_(*P*) = 3*L*(*P*)/4 = 11.3 nm: (a) *F *= 0 kV/cm and (b) *F *= 20 kV/cm, have been considered. (**c**) The results appear for two values of the applied electric field with *P *= 0 and *α*_0_ = 0.

**Figure 6:**
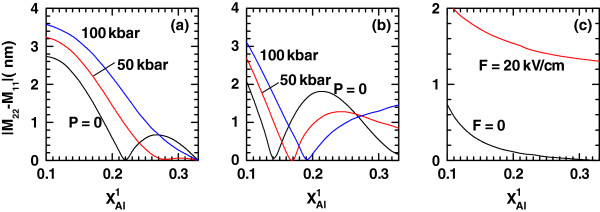
**The same as Figure**4**but for the absolute difference of dipole matrix elements|*M***_**22**_**−*M***_**11**_**|.**

In order to have a support for the discussion of the exciton-related nonlinear optical properties, Figures 4, 5, and 6 show, respectively, the variations of the transition energy $\mathrm{\Delta E}=E_{2}-E_{1}=E_{1}^{\text{e}}-E_{0}^{\text{e}}+E_{\text{b}}^{1,1}-E_{\text{b}}^{2,1}$ ( $E_{0}^{\text{e}}$ and $E_{1}^{\text{e}}$ are the energies of the ground and first-excited states, respectively, of the noncorrelated electron due to the confinement in the conduction band, and $E_{\text{b}}^{1,1}$ and $E_{\text{b}}^{2,1}$ are the binding energies in Figures 1 and 2, respectively), the transition dipole moment matrix element *M*_21_, and the absolute difference |*M*_22_−*M*_11_| as functions of $x_{\text{Al}}^{1}$. In all these figures, panel (a) corresponds to the zero-dc-field case for a fixed magnitude of the intense laser field strength *α*_0_=3*L*(*P*)/4 and the QD dimensions already adopted. The values of *P* used in previous figures are once again taken as curve parameters. The features exhibited by *ΔE*, *M*_21_ and |*M*_22_−*M*_11_| due to the growth in the left-hand Al molar fraction can be explained by the same type of arguments - related with the changes in the potential profile and wave function overlapping - given above and with the help of Figures 1 and 2. The most striking result is the vanishing of |*M*_22_−*M*_11_| for certain values of $x_{\text{Al}}^{1}$. This has something to do with the combination of a certain left-hand barrier height with the deformation of the potential band profile. There will be a particular configuration at which both exciton intrasubband matrix elements are equal in magnitude or both 0. This effect is reinforced by the action of the dc field, which makes it to appear at lower $x_{\text{Al}}^{1}$.

**Figure 7:**
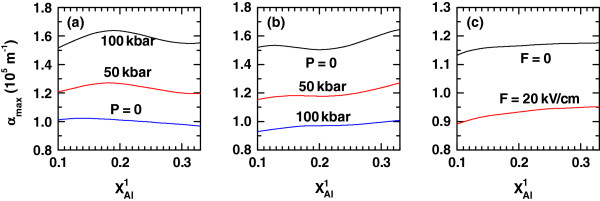
**Variation of the peak value of the exciton-related nonlinear optical absorption in a cylindrical QD.** The variation is a function of the aluminum concentration in the left-hand potential barrier ( $x_{\text{Al}}^{1}$). The results are for $x_{\text{Al}}^{2}=0.33$, *L *= 15 nm, and *R *= 10 nm. (**a**, **b**) Several values of the hydrostatic pressure with different setups of the applied electric field for *α*_0_=3*L*/4 = 11.3 nm: (a) *F *= 0 kV/cm and (b) *F *= 20 kV/cm, have been considered. (**c**) The results are for two values of the applied electric field with *P *= 0 and *α*_0_ = 0.

**Figure 8:**
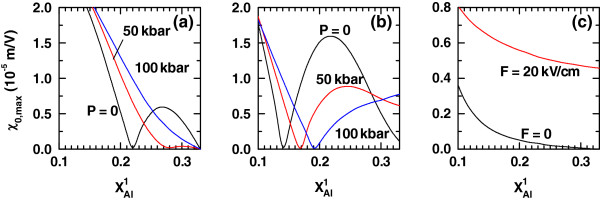
**The same as Figure**7**but for the resonant peak value of exciton-related nonlinear optical rectification.**

Figures 7 and 8 contain the evolution of the NOA and NOR resonant peaks due to the increment in the left-hand-barrier Al concentration. In the case of the absorption, one realizes that its variations, under the different circumstances considered in this work, are a direct consequence of the combination in the evolutions of *ΔE* and *M*_21_ as functions of $x_{\text{Al}}^{1}$ (see Equation 4). Even though the application of an axial dc field in a QD without applied intense laser implies a reduction in the amplitude of the NOA response at zero pressure (Figure 7c), the same situation but with an applied intense laser field results in the opposite effect. That is, with an intense laser acting on the QD, the application of an axially oriented electric field acts to enhance the resonant amplitude of the nonlinear absorption, which is only compared in magnitude to the non-laser case if the pressure becomes as high as 100 kbar. Therefore, the dc electric field is a tool for enhancing the optical absorption in cylindrical GaAs-Ga_1−*x*_Al_*x*_As QDs under intense laser radiation. Analogously, the shape of the dependence of the NOR coefficient on the variation of $x_{\text{Al}}^{1}$ follows mainly from the one corresponding to |*M*_22_−*M*_11_| according to Equation 5. By observing Figure 8c, we readily conclude that the cylindrical QD with an asymmetric axial potential barrier configuration at zero pressure and without the presence of static or intense laser electric fields is a rather worse optical rectifier if compared, for instance, with an asymmetric double QW [20], although the influence of a nonzero dc field slightly improves this property. If *F *= 0, but there is an intense laser applied, the tendency of the axial potential configuration to become symmetric causes the vanishing of the optical rectification coefficient, given the even and odd symmetries acquired by the ground and first-excited exciton states, respectively. In that case, the factor |*M*_22_−*M*_11_| identically vanishes because each of the two dipole matrix elements becomes equal to 0 (Figure 8a). The application of a dc field changes this situation given that, even if the Al concentrations in both barriers are the same, the presence of the linear dc field-related potential term prevents the carrier densities of probability to be symmetric (Figure 8b). The other values at which one detects the vanishing of the NOR correspond to potential configurations that, even with an asymmetric barrier profile, will present ground and first-excited probability density distributions that acquire a symmetry, thanks to the changes in the conduction and valence band profiles induced by the application of the intense laser field. As a result, the involved intrasubband exciton dipole matrix element may either simultaneously vanish, or they can become equal in magnitude, when these probability densities are integrated together with *z*_e_−*z*_h_.

## Conclusions

We have studied the cases of two 1*s*-like exciton complexes formed by the coupling of a ground-state hole with a ground-state electron and with an electron occupying the first-excited state in the axial motion of a GaAs-Ga_1−*x*_Al_*x*_As cylindrical quantum dot with symmetrical and asymmetrical potential energy profiles in the axial direction. Also, the amplitudes of the ground-to-excited exciton-related nonlinear optical absorption and nonlinear optical rectification coefficients are reported as functions of different external probes in the system, keeping a fixed geometric configuration for it, but changing the aluminum concentration in one of the axial potential barriers. Our work shows that hydrostatic pressure induces growth in both calculated binding energies. Increasing the Al concentration towards a symmetric axial potential profile configuration results in the reduction of the exciton binding energy in the zero-dc-field case, for given laser field intensities, whereas it results in the increase of this quantity when there is an additional static on-axis field applied, with the only exception of the first-excited exciton binding energy in the low-pressure regime.

The amplitude of the optical absorption resonant peak is a rather smooth function of the varying Al composition. The application of a dc field, in addition to the intense laser one, inverts the rate of variation of the optical absorption coefficient with respect to the increase in the hydrostatic pressure. The nonlinear optical rectification coefficient shows oscillations in its resonant peak amplitude due to the increment in the aluminum concentration of the left-hand axial barrier. In the zero-dc-field case, this amplitude can happen for the symmetric barrier profile case (equal Al composition in each of the barriers) and zero dc field or in cylindrical quantum dots with a particular axial left-hand-barrier Al concentration at which the effect of the laser on the shape of the confining potential causes the equality in the intrasubband exciton dipole matrix elements. This latter case can be present in the presence or absence of an applied dc electric field.

## Competing interests

The authors declare that they have no competing interests.

## Authors’ contributions

AZ carried out the numerical work. REA carried out the analytical work. MEMR carried out the discussion of results. CAD carried out the numerical work. All authors read and approved the final manuscript.
